# A Cluster Sleep-Wake Scheduling Algorithm Based on 3D Topology Control in Underwater Sensor Networks

**DOI:** 10.3390/s19010156

**Published:** 2019-01-04

**Authors:** Wenbo Zhang, Jing Wang, Guangjie Han, Xinyue Zhang, Yongxin Feng

**Affiliations:** 1 School of Information Science and Engineering, Shenyang Ligong University, Shenyang 110159, China; zhangwenbo@sylu.edu.cn (W.Z.); wang0113jing@163.com (J.W.); fengyongxin@sylu.edu.cn (Y.F.); 2 Key Laboratory for Ubiquitous Network and Service Software of Liaoning province, School of Software, Dalian University of Technology, Dalian 116024, China; 3 No. 2 Middle School of Shenyang, Shenyang 110006, China; zhang_xinyue@yeah.net

**Keywords:** underwater sensor networks, 3D topology control, clustering, cluster sleep-wake scheduling algorithm

## Abstract

3D topology control in underwater sensor networks is of great significance to ensuring reliable and efficient operation of the network. In this paper, by analyzing the characteristics of an underwater sensor network, we take the cube as the basic unit to perform 3D partition of the monitoring area, define the 3D partition unit and basic cluster structure of the underwater sensor network, and arrange rotating temporary control nodes in the cluster. Then, a cluster sleep-wake scheduling algorithm is proposed that compares the remaining node energy. It selects the node with the largest remaining energy as the working node, and the remaining nodes complete the transition of dormancy and waiting states as long as they reach the preset dormancy time. The node state settings of this phase are completed by the temporary control node. Temporary control nodes selecting and sleep-wake scheduling are used in the algorithm through 3D topology control, which reduces energy consumption and guarantees maximum sensing coverage of the entire network and the connection rate of active nodes. Simulation results further verify the effectiveness of the proposed algorithm.

## 1. Introduction

With the development of modern science and technology, human beings are more and more fully aware of the use and development of the environment, and the ocean, due to its rich resource reserves and research value, has prompted us to continuously explore the underwater space. The sensor nodes suitable for the underwater environment are deployed in the underwater environment for resource exploration, disaster prevention, data collection and other works. The sensor node forms a highly flexible underwater wireless sensor network (UWSN) through underwater acoustic communication. UWSN can be classified according to different standards: one is divided into static, semi-static and dynamic networks according to node mobility; another common classification is divided into 2D and 3D topologies structures according to whether the depth of nodes is considered [1].

In the common 2D UWSN structure, nodes are usually placed on the seabed [2]. Due to the special underwater environment, sensor nodes are small in size [3]. Unlike radio wave transmission, underwater signals can only be transmitted through sound channels, which will affect the communication quality [4]. In the 3D structure, nodes are distributed at different depths in the water to conduct comprehensive monitoring of the water area [5]. The depth of the sensor nodes can be controlled by buoys fixed on a horizontal plane [6]. The 3D network structure can more intuitively reflect the monitoring environment of underwater sensors [7]. However, the challenges of this structure involve nodes positioning and ensuring that all areas are continuously covered [8,9]. It is meaningless not to know the monitoring data of node location and if the area coverage is low, its application value will be affected. In addition, a good coverage method can also play a role in reducing the total cost [10]. Because the coverage of a single node is much smaller than that of target monitoring, many nodes must cooperate to achieve full target coverage [11]. Many algorithms optimize the coverage of the network from different angles [12], but this goal is still difficult to achieve, because the speed of water, the creatures in the water and the passing ships may destroy the placed nodes, thus affecting normal communication [13,14]. How to ensure network coverage in a complex underwater environment is a reliable and complete guarantee for UWSNs monitoring and collecting data.

The UWSNs sensor nodes are battery-powered and are often difficult to replace [15]. Network performance will be affected once the node energy is exhausted and the cover hole occurs [16]. Topology control enables the network to eliminate unnecessary communication links between nodes, equalize the energy of nodes in the network, and prolong the network lifetime on the premise of satisfying coverage and connectivity. Generally speaking, topology control mainly consists of topology structure and topology maintenance, and is one of the effective solutions to save energy and prolong lifetime [17]. By selecting the appropriate cluster head for the topology control of nodes in the network, the effect of balancing network energy consumption can be achieved [18]. The common topology control method is to set the sleep node [19]. In this paper, nodes distributed in water are divided into clusters, control nodes in the cluster are changed according to the residual energy of nodes, and the dormancy time of nodes is set in combination with the number of neighbors, so as to reduce network energy consumption and prolong network lifetime [20]. Based on the above background, this paper designs a cluster sleep-wake scheduling algorithm based on 3D topology control. The algorithm achieves high network coverage and connectivity, and the clustering and sleep-wake strategies greatly prolong the network lifetime.

The rest of this paper is organized as follows. Section 2 explains relevant research results on UWSNs and analyzes network energy consumption and coverage defects. Section 3 proposes a network model for UWSNs. Section 4 presents a cluster sleep-wake scheduling algorithm based on 3D topology control in UWSNs. Section 5 outlines the results of our experiments, and Section 6 concludes the paper.

## 2. Related Work

Compared with traditional WSNs, UWSNs, which uses an underwater acoustic channel for transmission, consumes more energy [21]. If the node runs out of energy, it will immediately fail, which will cause network coverage vulnerability and greatly affect network performance. Ref. [22] designed a microbial battery for sensor to solve the problem of limited energy. However, this way of collecting energy is affected by the environment, although it increases the survival time of nodes, it increases the cost. Therefore, saving sensor energy by controlling the behavior of nodes in UWSN is still a convenient and efficient way.

The routing algorithm PER (power-efficient routing protocol) and SEANAR (energy-efficient and topology-aware routing protocol) were compared in [23]. Both algorithms add location information and residual energy information of underwater nodes when selecting the next hop. PER algorithm consumes less energy and reduces the number of repeated packets sent, but it is prone to communication blanks and reduce network coverage. Based on topology control, SENAR finds and selects the next forwarding node in two stages, but the node near the sink has high energy consumption and is prone to energy holes. Khasawneh [24] et al. proposed a reliable and energy-efficient pressure-based routing (RE-PBR) algorithm. Considering the link quality, depth and residual energy, the information acquisition and routing and forwarding algorithm are designed to balance the energy consumption and make the routing protocol more efficient and energy-efficient. Because the algorithm does not consider the location of nodes, it will lose advantages in the perception and coverage of regions.

Considering the complexity of underwater environment, network stability, anti-interference and end-to-end delay must also be considered in the study of UWSNs [25]. In [26], an adaptive disjoint path vector algorithm suitable for heterogeneous wireless sensor networks was proposed. The algorithm has the characteristics of energy perception and distributed fault tolerance, including initial and recovery stages. The scheme can maintain the connection between nodes, but the advantage of optimizing energy consumption of nodes is not obvious.

The CLTC (Cluster-Based Topology Control) framework has been improved in [27]. First, the nodes were divided into clusters by k-means algorithm, and then topology control between clusters was implemented by using relative neighborhood graph. The greatest advantage of this algorithm was that it could maximize the network life cycle, but it offered no clear advantage in reducing inter-node interference. A method to solve the energy optimization problem of wireless sensor network was proposed in [28]. This method also involves topology control and multicast technology based on network coding. Compared with using them separately, this method improved overall performance. The scheme reduced end-to-end delay and optimized network energy consumption, but was slightly insufficient in network connectivity and coverage.

In [29], a basic theoretical algorithm based on WSNs was proposed. Topology control techniques of planar network and layered network were discussed respectively, and algorithms was further classified according to the key parameters of state scheduling. However, this algorithm has many shortcomings and needs to be further research.

Considering the two factors of interference and delay in topology control, three optimization goals were proposed in [30], maximum interference, average interference, and average path interference. A centralized algorithm with a greedy strategy was proposed that satisfied the delay constraint while minimizing interference as much as possible. Then, a distributed algorithm was proposed to reduce average interference. To minimize average path interference, a local delay constraint Bellman-Ford algorithm was also put forward. Finally, the network topology consisted of these optimal paths. The topology control algorithm showed good performance in anti-jamming and could guarantee end-to-end delay; however, the algorithm was insufficient in improving network coverage and extending the network life cycle.

Our contributions are as follows:

- In this paper, a cube is used as the partition unit, and a rule 3D space segmentation scheme is used for network partition. Based on the establishment of a 3D topological control model for UWSNs, the concepts of a UWSN unit and underwater cluster are proposed. The size of the partition unit and cluster scale are calculated according to the constraints of energy efficiency and quality-of-service awareness.
- A sleep-wake scheduling algorithm for UWSN clusters is constructed, which can build initial randomly placed sensors into a 3D network and form a UWSN with higher coverage, higher connectivity, and lower energy consumption through the sleep-wake scheduling algorithm.

## 3. Model of Underwater Wireless Sensor Network

**Figure 1:**
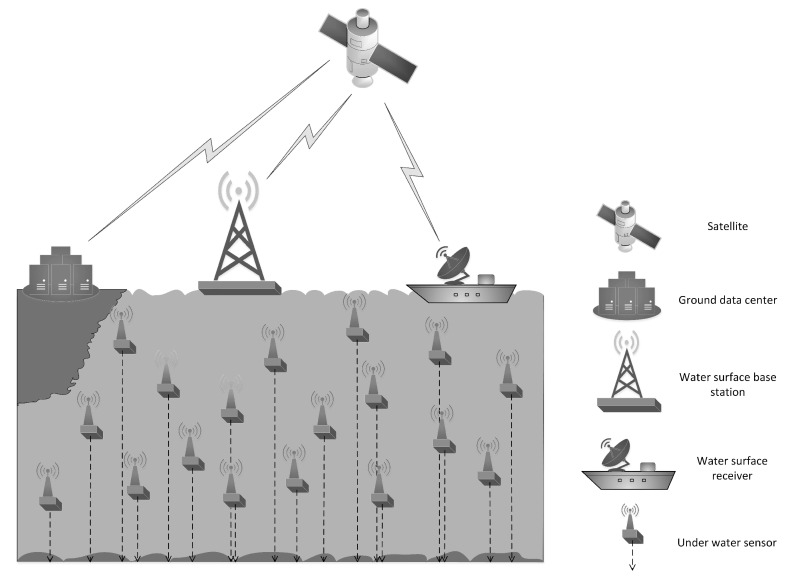
3D model of underwater wireless sensor network.

A 3D UWSN is used to detect and observe situations that cannot be fully observed through a submarine sensor node (i.e., for collaborative sampling work in a 3D marine environment). In a 3D underwater network, sensor nodes are suspended at different depths in the water for detection. In this structure, each sensor node is anchored to the bottom of the sea and equipped with buoys that can be inflated with pumps, as shown in Figure 1. The buoys can push sensors to the surface. The depth of a sensor can be adjusted by adjusting the length of the cord connecting the sensor and the anchor via an electric motor mounted on the sensor. Each sensor network has a surface receiver for collecting data and a surface base station for collecting, processing, integrating, and transmitting information.

**Figure 2:**
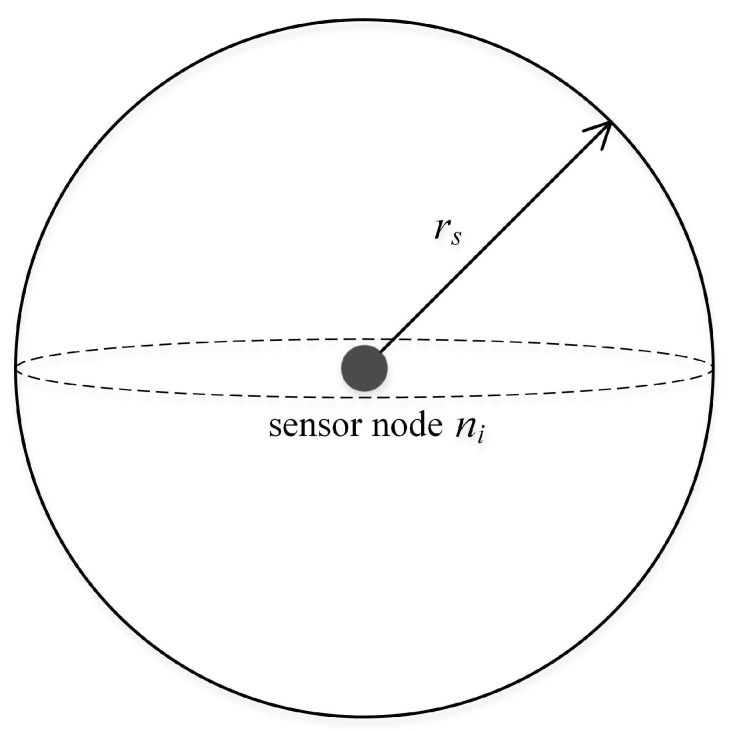
Boolean sensing model.

The sensor nodes in this paper use the 3D Boolean sensing model. Assuming that the coordinates of underwater sensor node $n_{i}$ in the 3D coordinate system of the monitoring area is $\left(x_{i},y_{i},z_{i}\right)$, the sensing radius is $r_{s}$. Therefore, the sensor node $n_{i}$ sensing model is a sphere with a spherical center $\left(x_{i},y_{i},z_{i}\right)$ and a radius $r_{s}$, labeled a sensing ball in Figure 2. For sensor node $n_{i}$, when the target object is in the sensing ball of $n_{i}$, then the target can be sensed by; if it is outside the sensing ball, $n_{i}$ cannot detect this target object.

We set the coordinates of any point *Q* in the 3D coordinate system of the monitoring area as $\left(x_{q},y_{q},z_{q}\right)$. The Euclidean distance from point *Q* to sensor node $n_{i}$ is $d\left(n_{i},Q\right)=\sqrt{{\left(x_{q}-x_{i}\right)}^{2}+{\left(y_{q}-y_{i}\right)}^{2}+{\left(z_{q}-z_{i}\right)}^{2}}$; therefore, the probability that the sensor node $n_{i}$ can perceive point *Q* is shown in Equation (1).

(1) $$\begin{array}{c} P\left(n_{i},Q\right)=\{\begin{array}{cc} 1, & d\left(n_{i},Q\right)\leq r_{s}\\ 0, & d\left(n_{i},Q\right)>r_{s} \end{array} \end{array}$$

**Figure 3:**
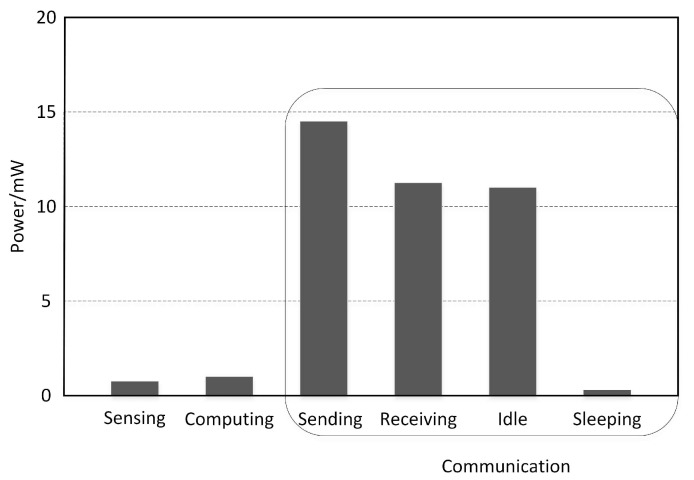
Energy consumption of sensor components.

According to the sensor node composition, wireless sensor sensing modules that consume energy consist of the module, calculation module, and wireless communication module. The energy consumption of each part of the node is presented in Figure 3. The wireless communication module consumes most energy, whereas the sensor node’s sensing module and calculation module consume less. Wireless communication modules are usually divided into four states: sending, receiving, idle, and sleep. Among them, energy consumed in the sending state is the highest of the four, energy consumed in the receiving state and the idle state is moderate, and energy consumed in the sleep state is minimal. When the node is idle, it must also monitor the channel constantly to determine whether data are being sent to itself; thus, it consumes energy. In the sleep state, the node turns off the sensing and communication modules, greatly reducing energy consumption.

In this paper, the energy consumption model only considers energy consumption when sending data, receiving data, and when in an idle state.

### 3.1. Energy Consumption for Sending Data

WSNs adopt energy consumption models based on multipath transmission and free space. Energy consumed by a node when sending and receiving data packets is related to the transmission distance, data packet size, and communication environment. A UWSN uses underwater acoustic signals to communicate, which produces a qualitative change compared to radio signals. Therefore, the energy consumption of underwater transmission should be reconsidered.

Due to the uniqueness of the underwater environment, the problem of data transmission has been transformed into a problem of signal attenuation in underwater acoustic channels in many underwater acoustic communication studies. Energy is consumed when sending data is determined by the packet size, minimum transmission power, and power attenuation function of the data.

Assuming that the sensor node can normally receive 1 bit data, the minimum power is $P_{min}$, and the power attenuation function with changing in transmission distance *d* is A(d).

Of these, the power attenuation function A(d) relates to attenuation coefficient α, transmission distance *d*, and the underwater acoustic channel transmission model is as follows:

(2) $$\begin{array}{c} A\left(d\right)=\alpha^{d}\times d^{k} \end{array}$$

where *k* represents the energy diffusion factor of the underwater acoustic channel. When k=1, the hydroacoustic channel adopts the cylindrical diffusion model; while k=1.5, the underwater acoustic channel adopts the spherical diffusion model; when k=2, the actual situation is indicated.

Usually, the attenuation coefficient α is directly related to the absorption coefficient α(f) as in the following relationship:

(3) $$\begin{array}{c} \alpha =10^{\frac{\alpha (f)}{10}} \end{array}$$

wherein, the unit of absorption coefficient α(f) is dB/m, which can be expressed as:

(4) $$\begin{array}{c} \alpha \left(f\right)=0.11\times \frac{10^{-3}\times f^{2}}{1+f^{2}}+44\times \frac{10^{-3}\times f^{2}}{4100+f^{2}}+2.75\times 10^{-7}\times f^{2}+3\times 10^{-6} \end{array}$$

where *f* is the unit *k* Hz, which represents the carrier frequency of the underwater acoustic signal.

Therefore, the energy required to send *n* bit data to another node at a distance of *d* meters in a shallow water area is:

(5) $$\begin{array}{c} E_{s}=n\times P_{min}\times A\left(d\right) \end{array}$$

(6) $$\begin{array}{c} E_{s}=n\times P_{min}\times 10^{d\times (0.011\times \frac{f^{2}}{1\;+\;f^{2}}+4.4\;\times \;\frac{f^{2}}{4100\;+\;f^{2}}+2.75\;\times \;10^{-5}\;\times \;f^{2}\;+\;0.0003)}\times d \end{array}$$

### 3.2. Energy Consumption for Receiving Data

The energy required to receive data is related to the packet size and energy consumed when receiving 1 bit data. In general, the constant $E_{elec}$ is used to represent energy consumed by the node when it receives 1 bit data. Therefore, energy consumption when receiving *n* bit data is:

(7) $$\begin{array}{c} E_{r}=n\times E_{elec} \end{array}$$

### 3.3. Energy Consumption in Idle Time

When idle, the node listens to the channel all the time, and no data are sent or received; thus, the energy consumption of the node at this time is related to the waiting time. Assume that the energy consumed by the sensor node listening to the channel is a constant $E_{m}$ per unit time. Therefore, when the node is idle for $t_{w}$ seconds, the energy consumption of the sensor is

(8) $$\begin{array}{c} E_{l}=t_{w}\times E_{m} \end{array}$$

### 3.4. Coordinate System

**Figure 4:**
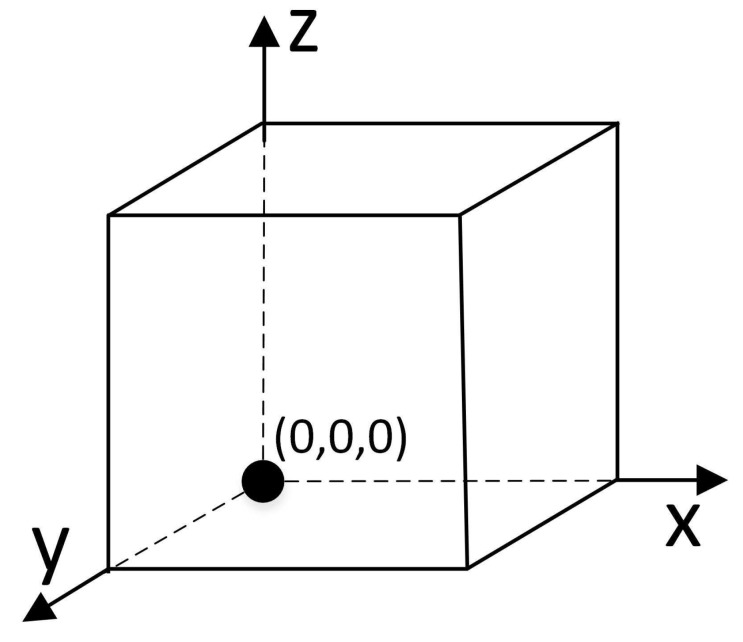
3D coordinate system for the monitoring area.

When studying coverage problems, the location information of the node is an absolute coordinate, leading to disadvantages in unified node management. In this paper, a coordinate system is established in the monitoring area, and the origin coordinate is specified; accordingly, the sensor node can obtain corresponding relative coordinates, rendering completion of the algorithm highly convenient. This paper takes the vertex in the lower-left corner of the monitored 3D region as the origin and establishes the 3D coordinate system for the monitored region, as shown in Figure 4.

### 3.5. Topological Model of 3D Dense Network

**Figure 5:**
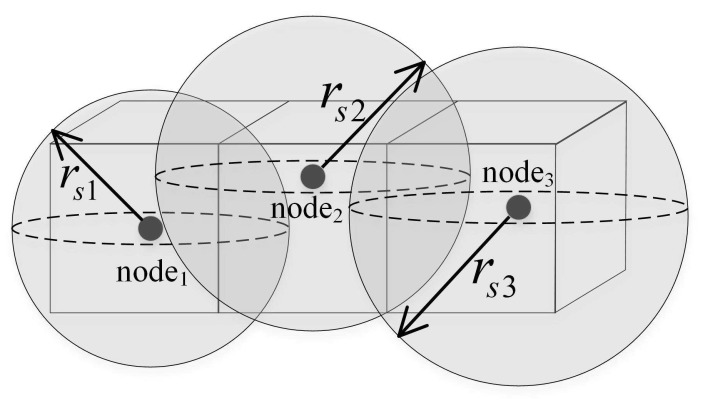
Topological model of three-dimensional dense network.

The topology control problem of 3D dense networks assumes that there are a large number of redundant nodes in the network, and these nodes are evenly and densely placed in the 3D space area to be monitored. The topological model of a 3D dense network divides the entire 3D space into multiple identical virtual components, guaranteeing an active node in each virtual unit at any moment; see Figure 5.

Because the position of the sensor node in the virtual component unit is not fixed and the sensor radius of the node itself is uncertain, the coverage rate can be controlled by these two parameters. This key issue is addressed in this paper.

## 4. Cluster Sleep-Wake Scheduling Algorithm

### 4.1. Related Definitions

**Definition** **1** (Coverage rate *C_r_*)**.** *The coverage rate of sensor networks refers to the ratio between the intersection of sensor nodes’ sensing range and the volume of the monitoring area, namely $C_{r}=\frac{V_{1}⋂V_{2}⋂\ldots ⋂V_{n}}{V_{A}}$.*

Where $V_{1},V_{2},\ldots ,V_{n}$ represents the sensing range of $node_{1}$, $node_{2}$, ..., $node_{n}$. $V_{A}$ refers to the volume of monitoring area *A*.

The coverage rate of a sensor network is an important indicator in evaluating the coverage performance of nodes; it represents the network’s sensing ability in the monitoring area but is not the only parameter by which to measure coverage performance. Therefore, coverage control for 3D UWSNs does not necessarily pursue a coverage rate of 1; rather, it should also consider the manufacturing and deployment costs of nodes and determine coverage requirements according to specific applications.

**Definition** **2** (Partition unit)**.** *A special polyhedron that can closely fill a 3D space without overlap or vacancy. The target 3D region A can be divided into several identical polyhedral $P_{u}$, as $V_{A}=\sum V_{P_{u}}$.*

**Figure 6:**
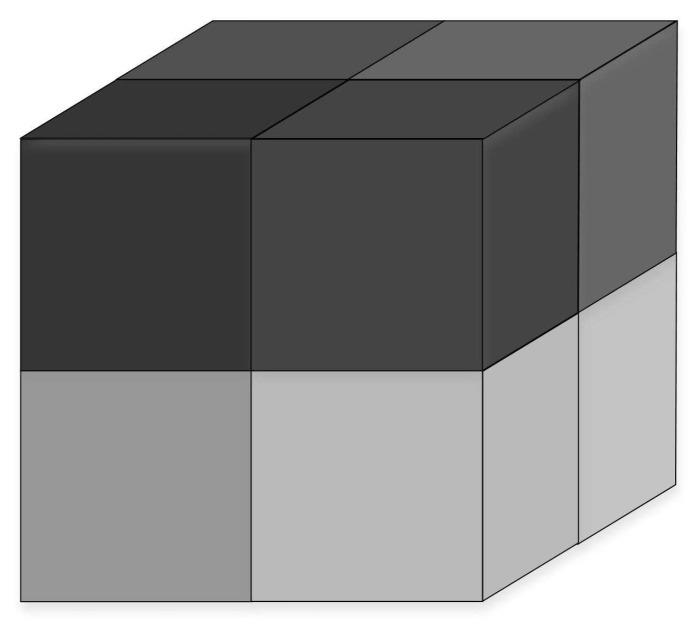
Cube consisting of 8 partition units.

The partition unit defined here is the virtual component unit mentioned above; therefore, the topology control of an underwater 3D network seeks to ensure an active node in each division unit at any time while maintaining high coverage. Cubes, hexagonal prisms, rhombic dodecahedrons, and truncated octahedrons can be used as segmentation units. Considering the convenience of subsequent algorithms, a cube is used as the partition unit in this paper. A regular 3D spatial segmentation scheme was adopted to divide the network, and the model is denoted as $CB_{d}$. Figure 6 depicts a cube consisting of eight partition units.

**Figure 7:**
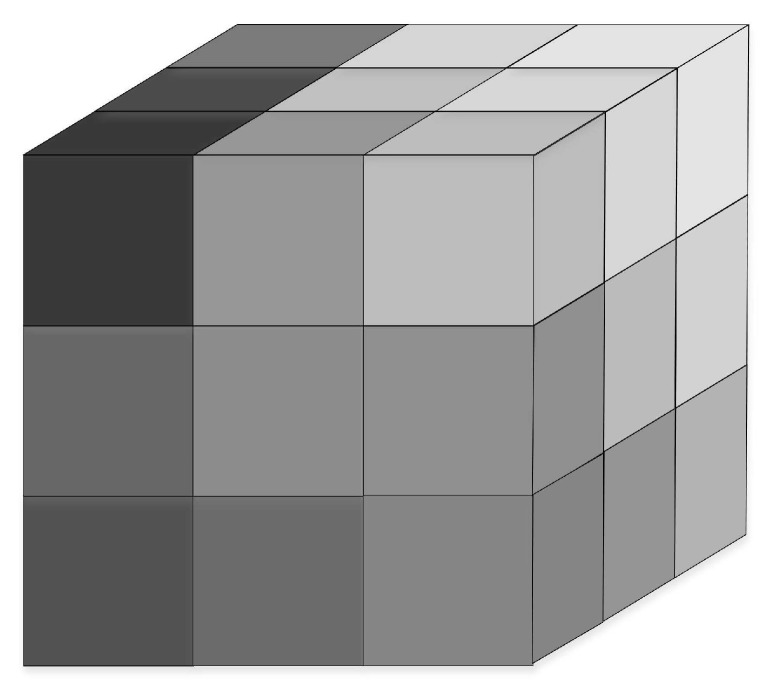
A cluster.

**Definition** **3** (Cluster)**.** *Each cube partition unit has 26 first-level physically adjacent units. Among them, six overlap with a surface of the segmentation unit and are called Class 1 adjacency units. Twelve adjacency units overlap with an edge of the partition units and are called Class 2 adjacency units. The eight adjacent units are overlapped with a point of the segmentation unit and are called Class 3 adjacent units. The cube, composed of the cube partition units and its 26 first-level physical adjacent units, is called a cluster as shown in Figure 7.*

**Figure 8:**
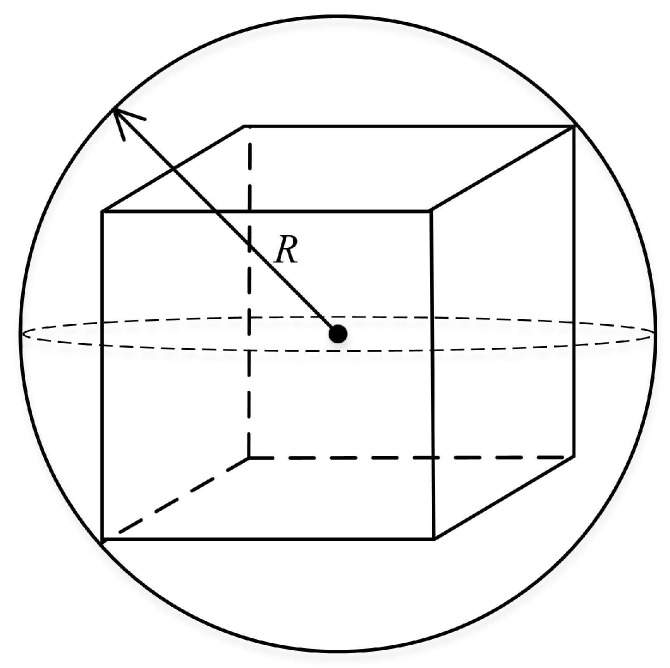
Partition unit and its radius.

**Definition** **4** (Partition unit radius *R*). *The radius of the partition unit refers to the radius of the circumscribed sphere of the partition cube, as shown in Figure 8.*

**Definition** **5** (Partition unit side length *l*)**.** *The partition unit side length l refers to the length of the side of the partition cube.*

**Definition** **6** (Cluster border length *L*)**.** *Similar to the definition of the partition unit length, it refers to the side length of a cluster. That is, L=3l.*

**Definition** **7** (Node number *ID*_0_)**.** *Set the coordinates of the nodes in the 3D coordinate system of the monitoring area as (x,y,z). Where z represents the length of rope between the node and the anchor and is a fixed value known at the beginning of deployment. The coordinates are the relative coordinates of sensor nodes with respect to the origin of coordinates, which can be obtained by the localization algorithm. Therefore, this unique coordinate is used in this paper as the node number, denote as $ID_{0}=\left(x,y,z\right)$.*

**Definition** **8** (Partition unit number *ID*_1_)**.** *For the convenience of partition unit management. A unique identification number is set for each partition unit in this paper, that is, partition unit number $ID_{1}$. Suppose the partition unit number $ID_{1}=\left(i,j,k\right)$, where i represents the number of rows in the partition unit, j represents the number of columns in the partition unit, and k represents the number of layers in the partition unit. Therefore, the coordinate range of each partition cube (i,j,k) can be obtained according to the partition unit number:*

(9) $$\left\{\begin{array}{c} (j-1)\times l\leq x<j\times l\\ (i-1)\times l\leq x<i\times l\\ (k-1)\times l\leq x<k\times l \end{array}\right.$$

Therefore, according to Equation (9), the node can get the number of the partition unit number through its number $ID_{0}$:

(10) $$\left\{\begin{array}{c} i=\lceil \frac{y}{l}+1\rceil \\ j=\lceil \frac{x}{l}+1\rceil \\ k=\lceil \frac{z}{l}+1\rceil \end{array}\right.$$

**Definition** **9** (Partition unit number *ID*_2_)**.** *For the convenience of cluster management, we also set up a unique identity number for each cluster in this paper, that is, cluster number $ID_{2}$. A detailed description will be given below. By the cluster definition, a cluster is comprised by 27 partition units. We have assumed that the cluster number $ID_{2}=\left(a,b,c\right)$. Then, the coordinate range of each cluster (a,b,c) can be obtained according to the cluster number:*

(11) $$\left\{\begin{array}{c} (b-1)\times L\leq x<b\times L\\ (a-1)\times L\leq x<a\times L\\ (c-1)\times L\leq x<c\times L \end{array}\right.$$

Therefore, according to Equation (11), node can get the cluster number by its own number $ID_{0}$:

(12) $$\left\{\begin{array}{c} a=\lceil \frac{y}{L}+1\rceil \\ b=\lceil \frac{x}{L}+1\rceil \\ c=\lceil \frac{z}{L}+1\rceil \end{array}\right.$$

**Figure 9:**
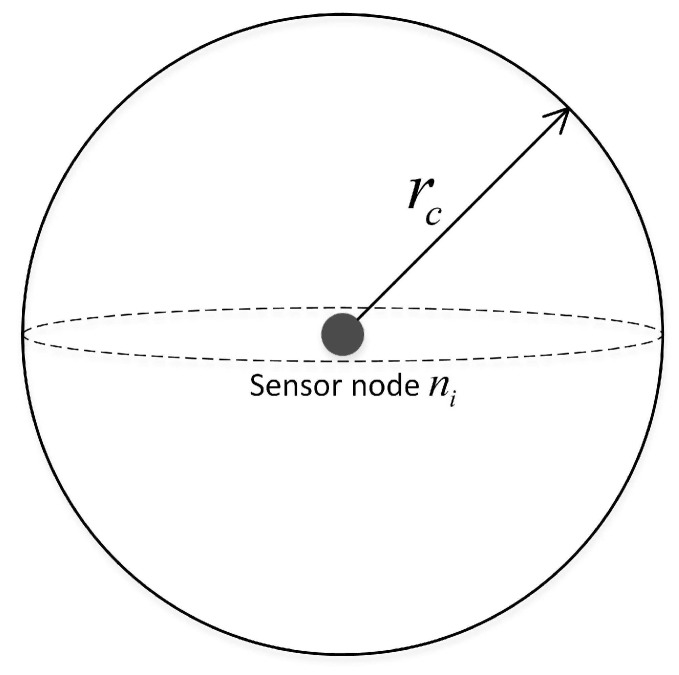
Communication range of sensor nodes.

**Definition** **10** (Communication radius *r_c_*)**.** *The communication range of the sensor node is similar to the sensing range. It is assumed that the coordinates of the underwater sensor node $n_{i}$ in the 3D coordinate system of the monitoring area are $\left(x_{i},y_{i},z_{i}\right)$, and the communication radius is $r_{c}$. Therefore, the sensor model of sensor node $n_{i}$ is a sphere with $\left(x_{i},y_{i},z_{i}\right)$ as the center of the sphere and $r_{c}$ as the radius, which can also be called a communication ball, as shown in Figure 9.*

**Definition** **11** (Node rank *R_ank_*)**.** *From the topological model of the 3D dense network mentioned above, it can be seen that, we can control the coverage rate by the two variable parameters of node location and sensing radius. In this paper, the node level is divided according to the location information of the node in the partition unit, denoted as the node rank $R_{ank}$. The specific division method and the corresponding sensing radius setting will be described in detail below.*

### 4.2. Related Work

#### 4.2.1. Determine the Size of the Partition Unit and Cluster

Partition units and clusters have been defined, and their size determines network quality; thus, it is necessary to determine the size of partition units and clusters to achieve high coverage, high connectivity, and low energy consumption throughout the network.

This study examines a 3D underwater environment, which is more complicated than the 2D land environment. Therefore, to manage sensor nodes more conveniently, a distributed method is adopted. In a 2D environment, cluster control is used for distributed control. In this paper, we simulate the clustering model in a 2D environment and divide the large-scale 3D region into a small 3D region by clustering. Each cluster exists independently, and the sensor scheduling problem is handled by temporary control nodes in the cluster.

It can be known from the cluster division that the temporary control nodes in the cluster can exist anywhere in the cluster. Therefore, to achieve single-hop communication within the cluster, the cluster size must be strictly controlled. According to the definition, the length of the cluster is, and it is assumed that the sensor nodes are distributed anywhere in the central plane of the splitting unit, and the farthest distance of the sensor nodes in the cluster is $\sqrt{22}l$. If you want to make all the nodes in the cluster can communicate in a single hop, then you must guarantee $r_{c}=\sqrt{22}l$, so the partition unit has a side length of $l=\frac{r_{c}}{\sqrt{22}}$ and the cluster side length is $L=\frac{3\times r_{c}}{\sqrt{22}}$.

#### 4.2.2. Determining Node Level and Sensing Radius

**Figure 10:**
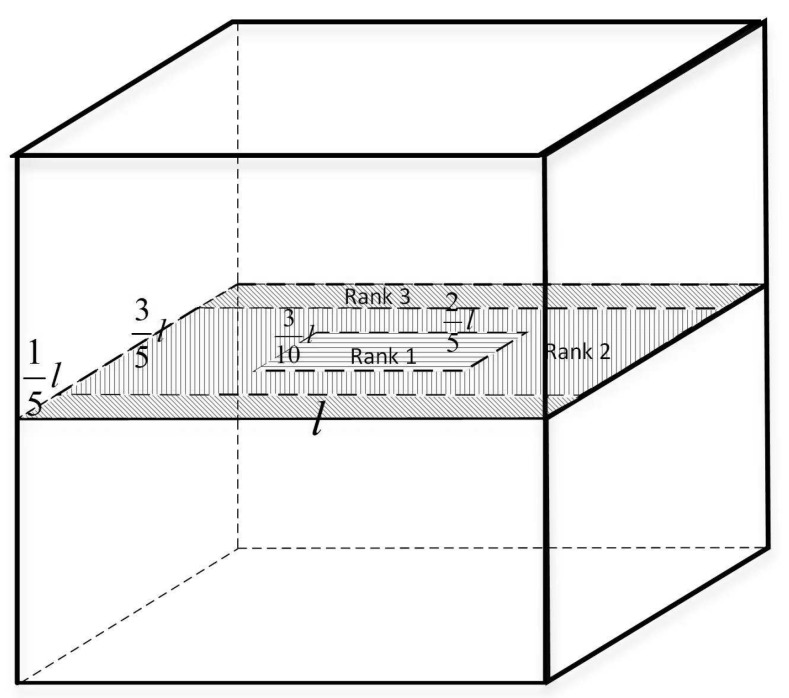
Node grading diagram.

Suppose an active node can be placed anywhere in the center plane of the partition unit, and it must be able to monitor any point in the partition unit. Therefore, the farthest distance that the sensor node in each split unit can measure is $\frac{3}{2}l=\frac{3\sqrt{22}}{44}r_{c}$, that is, the perceived radius of the node can be set to $r_{s}=0.32r_{c}$. However, according to the cluster size, the cluster side length is only $L=\frac{3\times r_{c}}{\sqrt{22}}=0.64r_{c}$. If the sensing radius is set in this way, only a large amount of overlay overlap will be caused. At the same time, in the monitoring, when the sensing radius is larger, the filtering and signal processing methods need to be more complicated, so that the required credibility can be achieved, which will inevitably increase the energy consumption. Therefore, under the condition that the sensing radius can be set, different sensing radii can be set for different node levels to reduce the redundant coverage under the premise of ensuring high coverage, thereby optimizing the sensing energy. Since the length of the anchor chain, that is to say the vertical height of the node, can be determined, it can be guaranteed that the node is always at the center plane of the partition unit. Therefore, the 3D coverage can be converted into 2D coverage, the node level is determined according to the 2D coordinates of the node, and the sensing radius corresponding to the nodes of different levels is obtained under the premise of ensuring high coverage, as shown in Figure 10.

(1) Primary nodes

As indicated in Figure 10, when the node is in the horizontal line area of the center plane of the partition unit, the node is a primary node; that is $R_{rank}$ = 1. At this point, the coordinate range of the node is:

(13) $$\left\{\begin{array}{c} x\in [\frac{\sqrt{3}}{9}\left(j-\frac{7}{10}\right)r_{c},\frac{\sqrt{3}}{9}\left(j-\frac{3}{10}\right)r_{c})\\ y\in [\frac{\sqrt{3}}{9}\left(i-\frac{13}{20}\right)r_{c},\frac{\sqrt{3}}{9}\left(i-\frac{7}{20}\right)r_{c})\\ z\in \forall \end{array}\right.$$

When the sensor node’s sensing radius is $r_{s}=\frac{\sqrt{3}}{2}l=\frac{1}{6}r_{c}$, the coverage rate can reach up to 100%.

(2) Secondary nodes

As shown in Figure 10, when the node is in the vertical line area of the center plane of the partition unit, the node is a secondary node; that is $R_{rank}$ = 2. At this point, the coordinate range of the node is:

(14) $$\begin{array}{c} \left\{\begin{array}{c} x\in [\frac{\sqrt{3}\left(j-1\right)}{9}r_{c},\frac{\sqrt{3}\left(j-\frac{7}{10}\right)}{9}r_{c})\\ y\in [\frac{\sqrt{3}\left(i-\frac{4}{5}\right)}{9}r_{c},\frac{\sqrt{3}\left(i-\frac{1}{5}\right)}{9}r_{c})\\ z\in \forall \end{array}\right.⋃\left\{\begin{array}{c} x\in [\frac{\sqrt{3}\left(j-\frac{3}{10}\right)}{9}r_{c},\frac{\sqrt{3}j}{9}r_{c})\\ y\in [\frac{\sqrt{3}\left(i-\frac{4}{5}\right)}{9}r_{c},\frac{\sqrt{3}\left(i-\frac{1}{5}\right)}{9}r_{c})\\ z\in \forall \end{array}\right.⋃\\ \left\{\begin{array}{c} x\in [\frac{\sqrt{3}\left(j-\frac{7}{10}\right)}{9}r_{c},\frac{\sqrt{3}\left(j-\frac{3}{10}\right)}{9}r_{c})\\ y\in [\frac{\sqrt{3}\left(i-\frac{4}{5}\right)}{9}r_{c},\frac{\sqrt{3}\left(i-\frac{13}{20}\right)}{9}r_{c})\\ z\in \forall \end{array}\right.⋃\left\{\begin{array}{c} x\in [\frac{\sqrt{3}\left(j-\frac{7}{10}\right)}{9}r_{c},\frac{\sqrt{3}\left(j-\frac{3}{10}\right)}{9}r_{c})\\ y\in [\frac{\sqrt{3}\left(i-\frac{7}{20}\right)}{9}r_{c},\frac{\sqrt{3}\left(i-\frac{1}{5}\right)}{9}r_{c})\\ z\in \forall \end{array}\right. \end{array}$$

When the sensor node’s sensing radius is $r_{s}=1.078l=0.23r_{c}$, the coverage rate can reach up to 100%.

(3) Tertiary nodes

As illustrated in Figure 10, when the node is in the oblique area of the center plane of the partition unit, the node is a tertiary node; that is, $R_{rank}$ = 3. The coordinate range of the node is:

(15) $$\begin{array}{c} \left\{\begin{array}{c} x\in [\frac{\sqrt{3}\left(j-1\right)}{9}r_{c},\frac{\sqrt{3}j}{9}r_{c})\\ y\in [\frac{\sqrt{3}\left(i-1\right)}{9}r_{c},\frac{\sqrt{3}\left(i-\frac{4}{5}\right)}{9}r_{c})\\ z\in \forall \end{array}\right.⋃\left\{\begin{array}{c} x\in [\frac{\sqrt{3}\left(j-1\right)}{9}r_{c},\frac{\sqrt{3}j}{9}r_{c})\\ y\in [\frac{\sqrt{3}\left(i-\frac{1}{5}\right)}{9}r_{c},\frac{\sqrt{3}i}{9}r_{c})\\ z\in \forall \end{array}\right. \end{array}$$

When the sensor node has a sensing radius of $r_{s}=\frac{\sqrt{6}}{2}l=0.26r_{c}$, the coverage rate can reach up to 100%.

#### 4.2.3. Handling Boundary Nodes

**Definition** **12** (Boundary region)**.** *A region consisting of all partition units which j=1 or $k=\lceil \frac{\sqrt{22}m}{r_{c}}\rceil$.*

**Figure 11:**
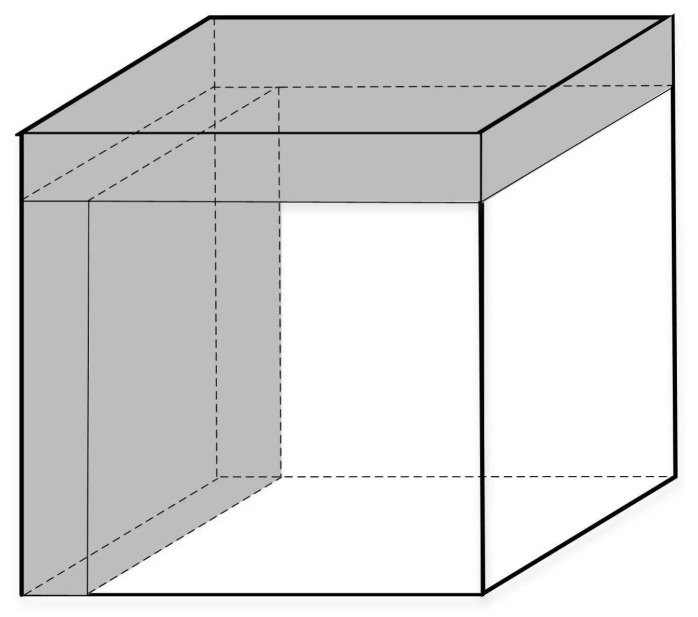
Boundary region.

Of these, *m* represents the height of the monitoring area. As shown in Figure 11.

**Definition** **13** (Boundary node)**.** *All sensor nodes are located in the boundary area.*

In an ocean monitoring network, due to the influence of tides, nodes deployed in the water must not be stationary. We must therefore consider the effect of the node’s movement on coverage. Because node movement does not produce a change in relative position, the coverage does not shift considerably; for most nodes, it is unnecessary to consider the effect of node movement on coverage. However, nodes at the boundary, including those at the left boundary and the upper boundary, must consider the impact of node movement on coverage. A specific sensing radius must thus be designed. A high coverage rate can be achieved even if the node leaves the partition unit.

**Figure 12:**
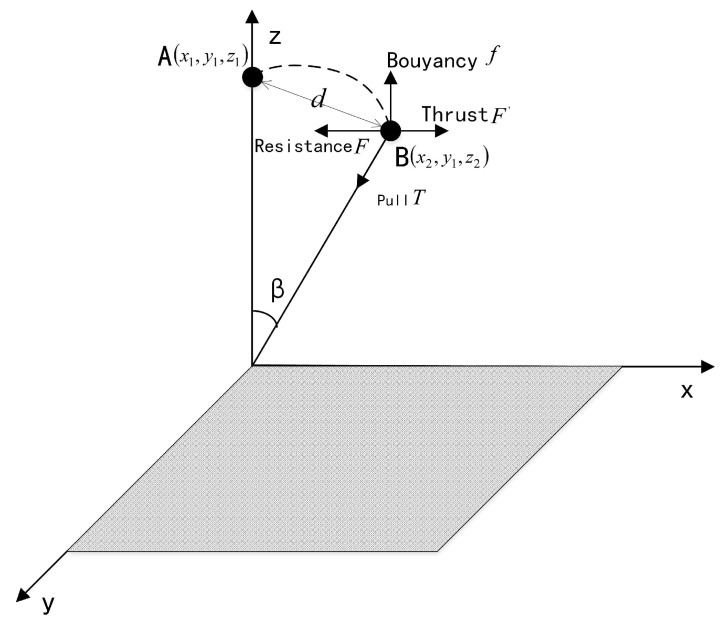
Stress and motion of nodes under water.

Assuming that the node is in a relatively stable ocean environment, the node will be thrust by the wave to the right, so the node only changes the coordinates on the *x*-axis and the *z*-axis during the motion. Since the nodes are pulled by the ropes, they do not move with the seawater to a distant distance position, but move within a certain range. As shown in Figure 12, it is the force of the node under water. According to this, the movement of the underwater node is a cyclical process. Therefore, the node moves along the curve from point A to point B, and then accelerates and then decelerates until the speed is zero. Randomly take a time $T_{p}$ in the time range $\left[T_{min},T_{max}\right]$ to describe the time when the node remains stationary at point B. Then use point B as the starting point to do the above movement. This cycle is the process of the node moving underwater. Because of the border node moves, there are coverage holes in the border area of the monitoring area. To solve this problem, it is necessary to reset the sensing radius of the boundary node.

It is assumed that the sensor node can obtain its own position coordinates according to the positioning algorithm under water. The coordinates of the node at the A position are $\left(x_{1},y_{1},z_{1}\right)$, and the coordinates of the node at the B position are $\left(x_{2},y_{1},z_{2}\right)$, as shown in Figure 12. Therefore, it can be known that when the moving speed of the node is zero, the horizontal distance $\left(d_{0}\right)$ and the vertical distance $\left(h_{0}\right)$ from the initial position are respectively.

(16) $$d_{0}=x_{2}-x_{1}$$

(17) $$h_{0}=z_{2}-z_{1}$$

Therefore, when the node is located in the partition unit of j=1, the perceived radius of the node is set to:

(18) $$r_{s}=r_{s}+d_{0}$$

When the node is located in the partition unit of $k=\lceil \frac{\sqrt{22}m}{r_{c}}\rceil$, the perceived radius of the node is set to:

(19) $$r_{s}=r_{s}+h_{0}$$

where $r_{c}$ represents the communication radius of the sensor node and *m* represents the water depth of the monitored water.

In addition, when j=1 and $k=\lceil \frac{\sqrt{22}m}{r_{c}}\rceil$, compare the size of $h_{0}$ and $d_{0}$, if $h_{0}>d_{0}$, the node sensing radius is set to:

(20) $$r_{s}=r_{s}+h_{0}$$

Conversely, the node’s sensing radius is set to:

(21) $$r_{s}=r_{s}+d_{0}$$

#### 4.2.4. Set Information Table

As subsequent node states change dynamically, it is necessary to know the relevant information of the node itself and within the cluster in advance. It is inconvenient to transfer this information individually; thus, this article includes an information table for nodes to be easily managed and transmitted; see Table 1.

### 4.3. Cluster Sleep-Wake Scheduling Algorithm

#### 4.3.1. Node Placement

**Figure 13:**
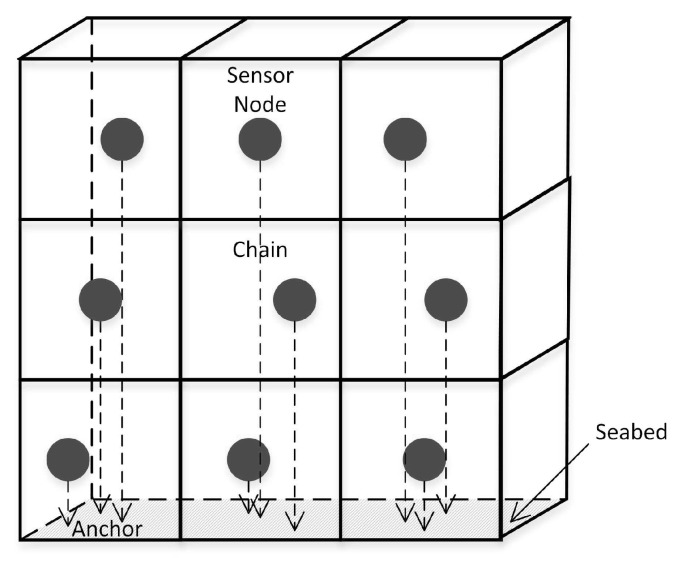
Node placement diagram.

Because of the 3D deployment, the 2D placement is converted into a 3D placement by changing the length of the rope connected to the anchor. When laying out, the nodes are laid out layer by layer. First, the nodes are randomly placed at the bottom of the water, and then the sensor is pushed to the surface by the buoy. The first rope length $l_{0}=\frac{\sqrt{22}}{44}r_{c}$, repeat the above action, the *n*th rope length is adjusted to $l_{0}=\frac{\left(2n-1\right)\sqrt{22}}{44}r_{c}$, where $0\leq n\leq \lceil \frac{\sqrt{22}m}{r_{c}}\rceil$, *m* represents the height of the monitoring area, as shown in Figure 13.

#### 4.3.2. Temporary Control Node Rotation

**Definition** **14** (Temporary control node)**.** *Each round of each cluster elects a temporary control node, which is equivalent to the cluster head node, responsible for controlling the dynamic changes of the nodes in the cluster, collecting the sensing information and sending it to the surface control node.*

Because the temporary control node has to do a lot of work, its corresponding energy consumption will be greater, so if you always use a temporary control node will cause it to die prematurely due to energy exhaustion, which is likely to cause coverage holes. Therefore, the rotation of the temporary control node is necessary.

Because the temporary control node is not only controlling the nodes in the cluster, it is also responsible for forwarding messages from other clusters. Therefore, the higher the number of node forwarding, the faster the corresponding energy consumption. Therefore, the rotation conditions of the temporary control node are adjusted according to the number of layers.

Therefore, $Currentenergy\leq \frac{Residualenergy}{c+1}$, which is $E\leq \frac{E_{residual}}{c+1}$, the node sends a temporary control node rotation message to the intra-cluster node, selects the next temporary control node, updates the remaining energy, and then enters the waiting state. If there is a temporary control node failure, the group broadcasts and a new temporary control node is elected.

#### 4.3.3. Set Hibernation Time

In this paper, three states are set for sensor nodes: the working state, waiting state, and deep dormant state as listed in Table 2.

**Figure 14:**
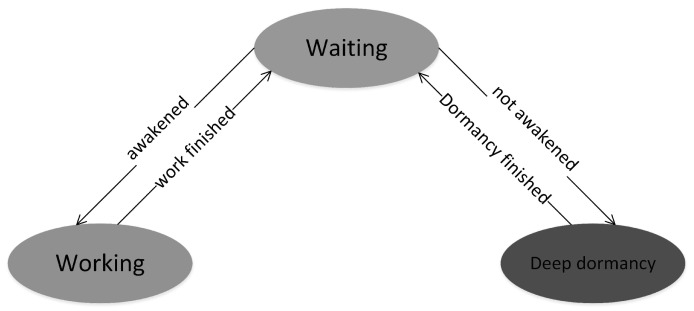
Node state conversion diagram.

The relationship between the three states is illustrated in Figure 14.

The relationship between the three indicates that the transition from the deep dormant state to the waiting state is controlled by the pre-set dormancy time. The dormancy time is set by the node timer. As soon as the time is right, the node transits to a waiting state. The longer the dormancy time, the smaller the energy consumption, but this will lead to no awakening node in the network. Therefore, considering network quality, more energy conservation is a problem to consider when setting the dormancy time in this paper.

The number of neighbors and remaining energy are important indicators determining the node life cycle in a partition unit; therefore, dual control conditions are used in this paper to set the dormancy time. A longer dormancy time for nodes results in fewer neighbors and less residual energy. Because the nodes closer to the water surface need to consume more energy, the dormancy time should be extended accordingly. Therefore, the dormancy time is

(22) $$T_{s}=\frac{c}{c+1}\cdot \left(1-\frac{num}{NUM}\right)\cdot \left(1-\frac{E_{residual}}{E_{n}}\right)\cdot t_{max}$$

where $t_{max}$ is the longest dormancy time, which must be set according to the actual situation.

Per the energy consumption model, assume that the energy consumed by the temporary control node per second is $E_{per}$, such that $E_{per}=E_{R}+E_{S}$.

From Equation (5),

(23) $$E_{s}=\lambda \cdot P_{min}\cdot A\left(d\right)$$

which represents the energy consumed by the temporary control node to send data per second. λ indicates that sensor node can send λ bit data per second, which is the transmission rate.

From Equation (7),

(24) $$E_{R}=\varepsilon \cdot E_{elec}$$

which represents the energy consumed by the temporary control node receiving data per second. ε indicates that the sensor node can receive ε bit data per second, denoting the receiving rate.

Here, $P_{min}$ represents the minimum power the sensor node can receive data normally; A(d) represents a power attenuation function with a communication distance of *d*. In this case, *d* refers to the communication radius $r_{c}$; $E_{elec}$ represents the energy consumed by receiving 1 bit data.

According to the temporary control node rotation conditions, when $E\leq \frac{E_{residual}}{c+1}$, the temporary control node will be re-selected. Given that $E_{residual}-t_{max}\cdot E_{per}\leq \frac{E_{residual}}{c+1}$, the maximum dormancy time can be set to

(25) $$t_{max}=\frac{c}{c+1}\cdot \frac{E_{residual}}{E_{per}}$$

#### 4.3.4. Description of the Algorithm

Step 1: Initial node placement.

Step 2: Select temporary control node. Each node broadcasts its own information table within the cluster and updates its information table based on the receiving message. Then, we select the partition unit within the cluster to make $\frac{num}{NUM}\cdot \frac{\sum_{num}E_{residual}}{\sum_{NUM}E_{residual}}$ largest. Finally, the largest node of $E_{residual}$ is found, making it the temporary control node of the cluster.

Step 3: Select working node. According to the node’s remaining energy $E_{residual}$, the temporary control node locates the node with largest $E_{residual}$ in each partition as the working node. Other sensors enter deep dormancy.

Step 4: State conversion. The node is not always in deep dormancy; it presets a dormancy time $T_{s}$ before dormancy. When it reaches the dormancy time, the sensor node automatically transitions from deep dormancy to a waiting state, awaiting the command from the temporary control node.

Step 5: Temporary control node rotation. When $E\leq \frac{c}{c+1}E_{residual}$, replace the temporary control nodes; then, return to Step 2.

Step 6: Repeat the above steps until the node energy in the WSN is zero. The detailed algorithm is shown in Algorithm 1.

**Algorithm 1** Cluster sleep-wake scheduling algorithm
**Input:** $ID_{0}$, $E_{n}$, $E_{residual}=E_{n}$, num=0, NUM=0, $t_{max}=\frac{1}{c+1}\cdot \frac{E_{residual}}{E_{per}}$, remaining energy in the network $E_{total}=0$;**Output:** Coverage rate $C_{r}$, energy consumption $E_{c}$, algorithm working time $T_{total}$;
  1:Calculate partition unit number $ID_{1}$, to which it belongs from Equation (10);  2:Calculate cluster number $ID_{2}$ to which it belongs from Equation (12);  3:Calculate node rand $R_{rank}$ from Equations (13)–(15). Set the sensing radius accordingly;  4:**while** 1 **do**  5:      Node in a waiting status, broadcasting message table in the cluster;  6:      **while** not traversed all nodes in the cluster yet **do**  7:            Find the same node of $ID_{1}$, record the number of nodes;  8:            num←num;  9:            Find the same node of $ID_{2}$, record the number of nodes;10:            NUM←NUM;11:      **end while**12:      Select the partition unit with the largest $\frac{num}{NUM}\cdot \frac{\sum_{num}E_{residual}}{\sum_{NUM}E_{residual}}$ within the cluster;13:      Select the node with the largest $E_{residual}$ in the partition unit as the temporary control node;14:      Temporary control node finds node with largest $E_{residual}$ in each partition as a working node;15:      Other remaining sensors enter deep dormancy;16:      Calculate coverage rate $C_{r}$ and dormancy time $T_{s}$;17:      Output $C_{r}$;18:      **while** not traversed all dormancy node yet **do**19:            **if** $t=T_{s}$ **then**20:               Node changes to waiting state;21:            **end if**22:            **if** $E\leq \frac{c}{c+1}E_{residual}$ **then**23:               Covert temporary control node to ordinary node;24:               Update $E_{residual}\leftarrow E_{residual}$, $t_{max}\leftarrow t_{max}$;25:            **end if**26:      **end while**27:      **while** not traversed all nodes yet **do**28:            Calculate $E_{total}=E_{total}+E$;29:      **end while**30:      **if** $E_{total}=0$ **then**31:            break;32:      **end if**33:      Calculate $E_{c}$;34:      Output $E_{c}$, $T_{total}$;35:
**end while**
36:Output $T_{total}$.


In this paper, initial node placement in the cluster sleep-wake scheduling algorithm is random. Then, a UWSN with high coverage, high connectivity, and low energy consumption is formed through scheduling between nodes. In the simulation experiment, compared with the general random deployment algorithm, the network coverage rate, connection rate, and network life cycle are important network evaluation indicators.

## 5. Simulation and Analysis

In this paper, the cluster sleep-wake scheduling algorithm is compared with the most common random deployment algorithm and the redundant node sleep algorithm based on sensing contribution. It is proved that the cluster sleep-wake scheduling algorithm can improve network coverage and save energy consumption. In the simulation, we set the size of the target water to 100 m × 100 m × 100 m. At the same time, because the cluster sleep-wake scheduling algorithm and the redundant node sleep algorithm are used to solve the problem of redundant nodes in the sensor network, it is necessary to increase the node redundancy in the network during the simulation. Therefore, we randomly placed 300 nodes in the target waters. The simulated environmental parameters are shown in Table 3.

Wherein, the node sensing radius of the cluster sleep-wake scheduling algorithm is related to the node level $R_{ank}$. While $R_{ank}=1$, the sensing radius is set to $r_{s}=14.43$ m; while $R_{ank}=2$, the sensing radius is set to $r_{s}=17.97$ m; while $R_{ank}=3$, the sensing radius is set to $r_{s}=20.41$ m. The sensing radius of the random deployment algorithm and the redundant node sleep algorithm is set to $r_{s}=20$ m.

**Figure 15:**
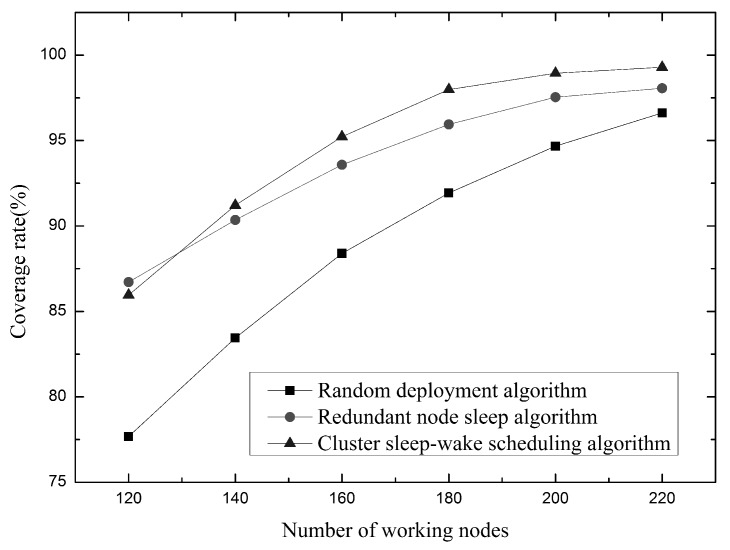
Relationship between the number of working nodes and coverage.

In Figure 15, the relationship between the number of working nodes and the coverage rate is shown under the same node communication radius and sensing radius. It can be clearly seen that the three algorithms are gradually increasing in network coverage as the number of working nodes increases. Although the network coverage of the redundant node dormancy algorithm will be higher when the number of working nodes is small, the network coverage of the cluster sleep-wake scheduling algorithm will be higher as the number of working nodes increases, and this trend will be keep going. This is because the cluster sleep-wake scheduling algorithm selects only one working node in each partition unit, which ensures that the nodes can be evenly distributed in the target waters, reducing node redundancy. The redundant node sleep algorithm selects the node with high perceived contribution as the working node, but there may be too many local nodes, which will increase the node redundancy. Therefore, the network coverage of the cluster sleep-wake scheduling algorithm is higher when the number of working nodes is the same.

**Figure 16:**
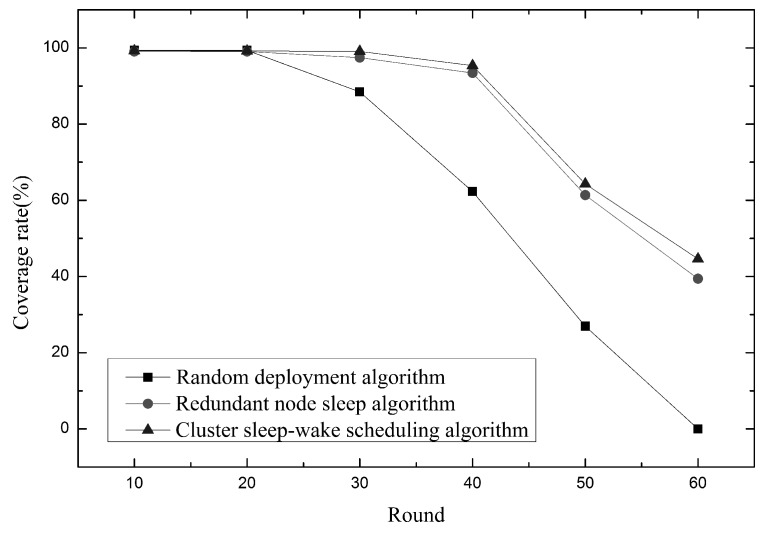
Relationship between network coverage and time.

As shown in Figure 16, it is the relationship between network coverage and time. At the beginning of the algorithm, 300 nodes are put into the network at the same time, all three algorithms can achieve high coverage. When entering the 30th round, the coverage of the random deployment algorithm is greatly reduced. Although the coverage change of the cluster sleep-wake scheduling algorithm and the redundant node sleep algorithm is small, it can be seen that the coverage of the cluster sleep-wake scheduling algorithm is higher. This is because the cluster sleep-wake scheduling algorithm adopts a node rotation strategy, and when the threshold is reached, the working node is replaced, so that the energy consumption can be balanced, and the working node exists in each partition unit as much as possible. The redundant node sleep algorithm selects to wake up the redundant node after the node energy is exhausted, and the sensing contribution of the redundant node is smaller than the perceived contribution of the working node, so the node perceptual contribution in the network increases with time. The degree is reduced and the node redundancy is increased. Therefore, the network sleep coverage of the cluster sleep-wake scheduling algorithm is higher when the runtime is the same.

**Figure 17:**
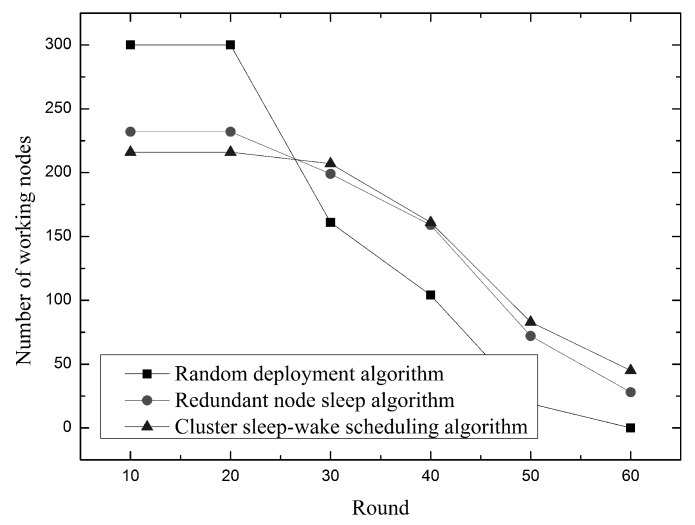
Relationship between the number of working nodes and time.

As shown in Figure 17, the change in the number of working nodes in the network is shown over time. In the case of deploying 300 nodes as well, the random deployment algorithm is that all nodes work at the same time, so that there will be a large drop in the number of working nodes over time. At the same time, the existence of a large number of redundant nodes will consume a large amount of energy, making the lifetime cycle of the network very short, and the entire wireless network will die in the 60th round. Both the cluster sleep-wake scheduling algorithm and the redundant node sleep algorithm adopt the redundant node sleep wake-up method. Therefore, only about 200 nodes are needed at the beginning of the network operation to achieve full coverage. The cluster sleep-wake scheduling algorithm uses a dense network topology model to ensure that there is one working node in each split unit, which can reduce node redundancy and increase node use. Therefore, there will be fewer working nodes in the cluster sleep-wake scheduling algorithm in the first 25 rounds. In the last 25 rounds, the number of working nodes in the cluster sleep-wake scheduling algorithm will be more. This is because the cluster sleep-wake scheduling algorithm divides the nodes in a cluster manner, uses the temporary control nodes to perform inter-cluster communication, reduces the number of data packet forwarding, and sets the sensing radius according to the node level to avoid unnecessary energy consumption, so the energy consumption in the network is There will be fewer, more nodes will work. Therefore, in the last 25 rounds, when the running time is the same, the cluster sleep-wake scheduling algorithm has more working nodes.

**Figure 18:**
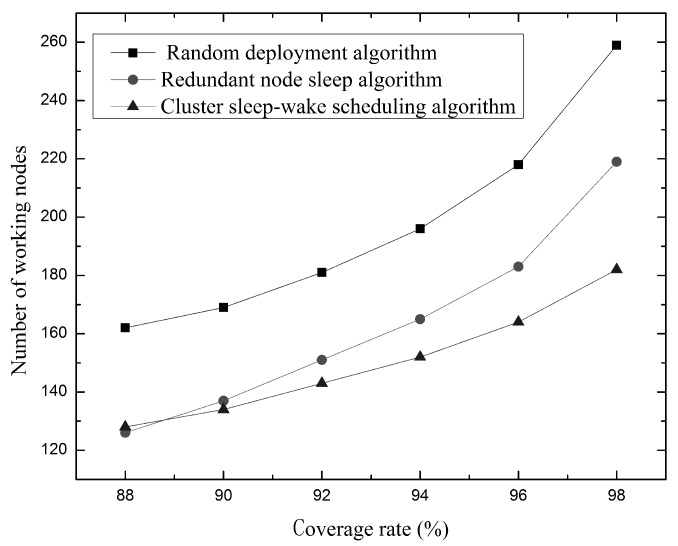
Relationship between coverage and the number of working nodes.

As shown in Figure 18, shows how the number of working nodes required changes when network coverage changes. Compared with the redundant node sleep algorithm, with the increase of network coverage, the growth trend of the number of working nodes required by the cluster sleep-wake scheduling algorithm is more gradual. At the same time, in order to achieve 98% network coverage, the random deployment algorithm may require about 260 working nodes, the redundant node sleep algorithm may need about 220 working nodes, and the cluster sleep-wake scheduling algorithm only needs about 180 working nodes. This is because the cluster sleep-wake scheduling algorithm uses the division of the splitting unit to ensure that the working nodes can be more evenly distributed in the sensor network and improve the use of the nodes. At the same time, when the node is awakened, the partition units with coverage holes are also selected, which can reduce node redundancy and greatly improve network coverage. Therefore, when the coverage ratio is the same, the cluster sleep-wake scheduling algorithm has fewer working nodes.

**Figure 19:**
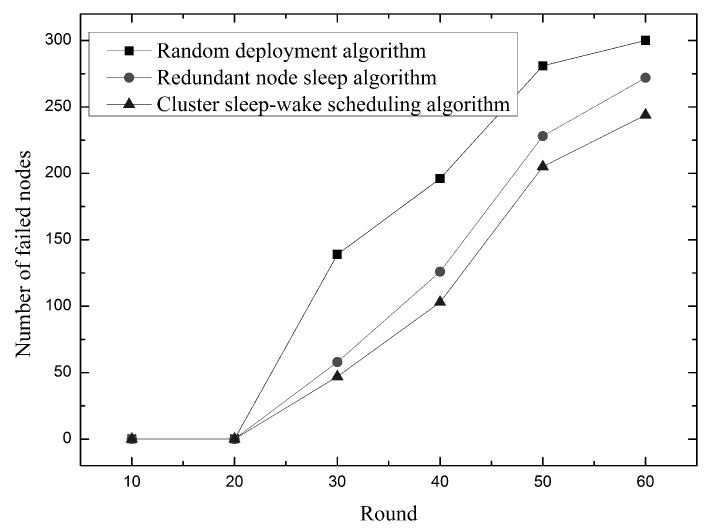
Relationship between the number of failed nodes and time.

As shown in Figure 19, shows how the number of failed nodes changes over time. As you can see from the figure, the failed node starts to appear around 20 rounds. Compared with the random deployment algorithm and the redundant node dormancy algorithm, the cluster sleep-wake scheduling algorithm has a lower number of failed nodes and a slower growth trend. This is because the cluster sleep-wake scheduling algorithm uses a sleep-wake policy and a node rotation strategy to balance energy consumption and prevent a node from rapidly dying. At the same time, the method of dividing the cluster is used to reduce the number of times of forwarding of the data packet, and the method of dividing the node level is used to reduce the excess sensing energy consumption. The redundant node sleep algorithm only considers the sleep-wake strategy, and chooses to wake up the redundant node after the dead node appears. Therefore, in the case of the same runtime, the cluster sleep-wake scheduling algorithm has fewer failed nodes.

**Figure 20:**
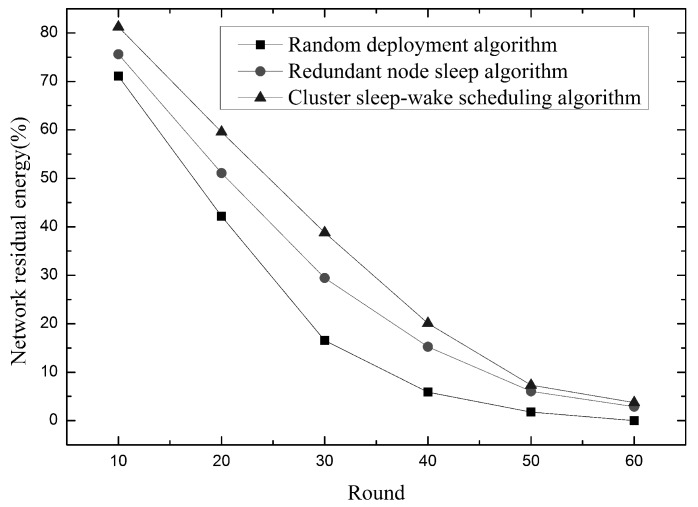
Relationship between network residual energy and time.

As shown in Figure 20, shows how the remaining energy in the network changes as the network runs longer. It can be seen from the figure that the network residual energy of the cluster sleep-wake scheduling algorithm is always higher than the other two algorithms during the network operation. This is because compared with the redundant node sleep algorithm, the cluster sleep-wake scheduling algorithm adds node rotation strategy, cluster partition mode and node classification mode to save energy consumption to reduce energy consumption. At the same time, since the perceived contribution of redundant nodes in the redundant node sleep algorithm is smaller than the perceived contribution of the working node, the more redundant nodes are awake, the greater the node redundancy in the network. Therefore, when the node fails, if you want to restore the network coverage, you need to wake up more redundant nodes, so there will be more working nodes in the network, so it will consume more energy. Therefore, when the network running time is the same, the cluster sleep-wake scheduling algorithm has more network residual energy.

**Figure 21:**
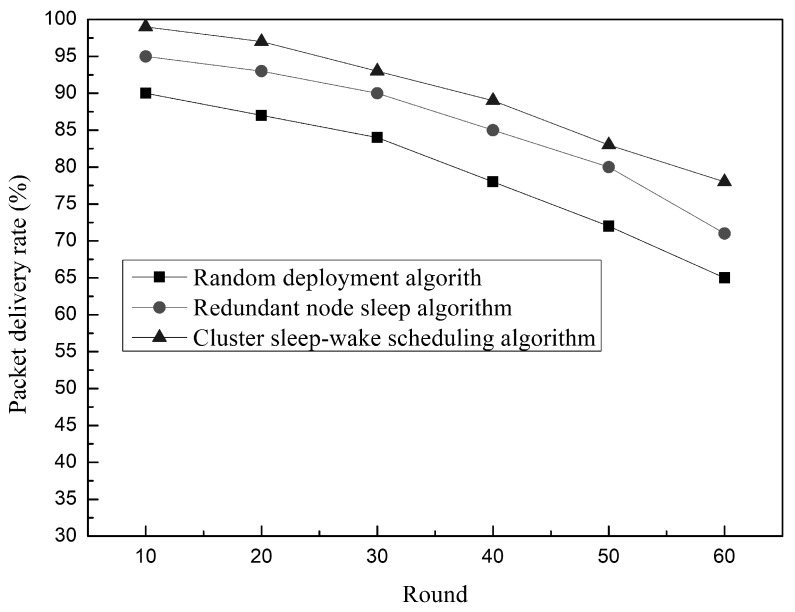
Relationship between the delivery rate and time.

The relationship between the delivery rate of UWSN network nodes and the number of experimental rounds is shown in Figure 21. In general, the delivery rates of the three algorithms tend to decrease with the increase of the number of rounds. This is due to the gradual death of nodes in the network, which affects the connectivity of the network. The random deployment algorithm and the redundant node sleep algorithm set a fixed sensing radius for the node, and the cluster sleep-wake scheduling algorithm sets the sensing radius of the node by the rank of the node. In addition, the algorithm ensures as much as possible that there are working nodes in each partition unit to keep the network connected. The different sensing radius settings of the nodes enable the network to maintain a higher connectivity in the same experimental environment. The rotation strategy of the control node of the cluster sleep-wake scheduling algorithm and the sleep wake-up method of the node reduce the energy consumption of the network and avoid premature death nodes, which provides a reliable condition for the network to maintain high connectivity. Therefore, under the same experimental conditions, the cluster sleep-wake scheduling algorithm has higher delivery rate than the random deployment algorithm and the redundant node sleep algorithm.

**Figure 22:**
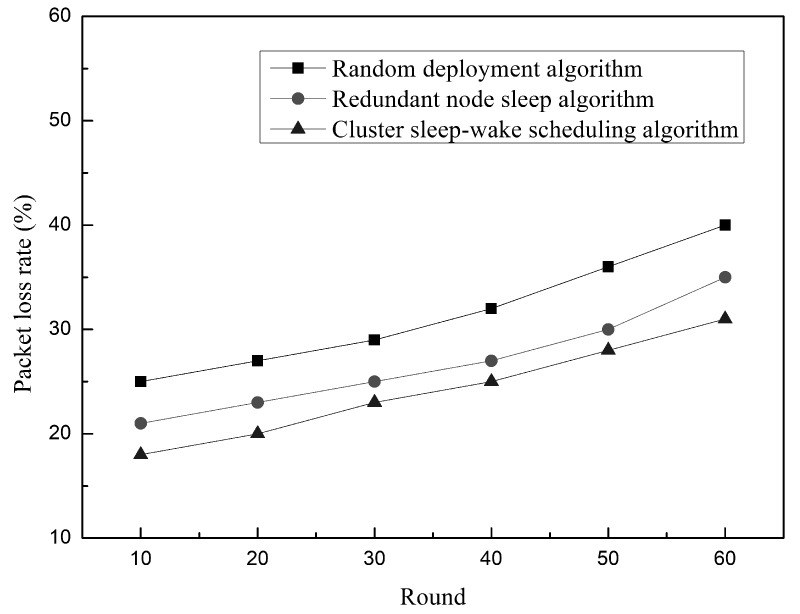
Relationship between the packet loss rate and time.

The relationship between the packet loss rate and the number of experimental rounds in the network is shown in Figure 22. It can be clearly seen that the packet loss rate of the three algorithms increases with the network operation. This is because the three algorithms use the same routing protocol (Vector-Based Forwarding Protocol (VBF)), so the packet loss situation increases. The trend is consistent. However, compared with the cluster sleep-wake scheduling algorithm, the random deployment algorithm and the redundant node sleep algorithm have higher packet loss rates when transmitting the same data packet (30 kpt/min). This is because the cluster sleep wake-up scheduling algorithm deploys the nodes, so that the underwater nodes are all within the communication range, ensuring reliable transmission of data. Further, they use the control node rotation strategy to prevent the control node from running out of energy and causing packet loss. All of the above methods can reduce the packet loss problem caused by the node itself in the process of data transmission during the cluster sleep-wake scheduling algorithm. Therefore, although the packet loss rate of the network increases with time, the cluster sleep-wake scheduling algorithm can still maintain a lower packet loss rate.

## 6. Conclusions

In this paper, a cluster sleep-wake scheduling algorithm is designed for an underwater 3D environment. In the initial stage of the algorithm, many sensor nodes were randomly placed in a 3D environment, and then the sensor network was divided into partition units and clusters. Temporary control nodes could prepare for subsequent distributed scheduling. Given the emergence of redundant nodes in the network, operations such as waking up working nodes, sleeping useless nodes, and rotating temporary control nodes were performed to save network energy consumption and extend the network lifetime cycle. Finally, a UWSN with good coverage, connectivity, and network lifetime cycle was obtained. Simulation experiments revealed that compared with random deployment, the proposed algorithm performed better in connectivity, coverage, and network lifetime cycle. Compared with the depth adjustment algorithm in the same underwater 3D environment, the algorithm also had advantages in coverage.

In UWSN, excellent topology control ensures network coverage and connectivity. Due to the complex underwater environment, using a 3D coordinate system to study UWSNs is realistic and has research value. The scheduling algorithm designed in this paper, based on the network’s 3D topology control, analyzes residual node energy and connectivity and sets the node’s sleep and wake time. Simulation results indicate that, under the same experimental conditions, the proposed algorithm has better coverage and connectivity than the random deployment algorithm. The sleep-wake mechanism of the working node in the algorithm can prolong the network life cycle. Simulation results also show that the cluster sleep-wake scheduling algorithm is more efficient compared with other similar topology control algorithms.

## Figures and Tables

**Table 1:** Node Information Table.

Parameter	Meaning
$ID_{0}$	Node location coordinate
$ID_{1}$	Partition unit number
$ID_{2}$	Cluster number
num	Number of nodes in partition unit
NUM	Number of nodes in cluster
$E_{n}$	Initial energy
$E_{residual}$	Surplus energy
$R_{rank}$	Node sink

**Table 2:** Three sensor states.

States	Sensing Module	Communication Module
Workstate	ON	ON
Waitingstate	OFF	ON
Deepdormantstate	OFF	OFF

**Table 3:** Environmental parameters.

Attribute Parameter	Value
examination range	100 m × 100 m ×100 m
Number of nodes	300
Sensing radius	14.43 m, 17.97 m, 20.41 m, 20 m
Partition unit side length	16.67 m
Cluster border length	50 m
Number of partition unit	216
Number of cluster	8
Initial energy of the node	100 J
$P_{min}$	$2\times 10^{-4}$ W
$E_{elec}$	$2\times 10^{-4}$ J
$E_{m}$	$2\times 10^{-3}$ J
